# Challenges and Prospects in Epilepsy Monitoring Units: A Comprehensive Review of Logistic Barriers

**DOI:** 10.7759/cureus.59559

**Published:** 2024-05-03

**Authors:** Anas Albarrak

**Affiliations:** 1 Department of Internal Medicine, College of Medicine, Prince Sattam Bin Abdulaziz University, Al-Kharj, SAU; 2 Department of Internal Medicine, College of Medicine, King Saud University, Riyadh, SAU

**Keywords:** patient-centered care, epilepsy monitoring unit, data safety and monitoring, epilepsy research, adult neurology

## Abstract

Epilepsy is one of the most common neurological diseases with a prevalence ranging from 0.5% to 2% in different sittings. The World Health Organization (WHO) estimated that nearly 80% of this burden is borne by resource-poor countries where even conventional electroencephalogram (EEG) coverage is dramatically short.

Video EEG monitoring applied for days as conducted in epilepsy monitoring units (EMUs) is aimed at seizure localization, anti-seizure medication (ASM) adjustment, or epilepsy surgery evaluation and planning. However, the EEG approach in EMUs has its obstacles. The present article is aimed to concentrate on the logistic challenges of EMUs, discussing existing data and limitations and offering suggestions for future planning to enhance the utilization of existing technology.

Shortages of adult and pediatric epileptologists, qualified nurses, as well as EEG technologists have been reported in different countries. Moreover, injuries and falls, psychosis, status epilepticus, and unexpected death have been stated to be the most frequent safety issues in EMUs. Enhancements to mitigate logistical and healthcare system-related barriers in EMUs include the implementation of large cohort studies and the utilization of artificial intelligence (AI) for the identification and categorization of specific risks among EMU admissions. The establishment of EMUs and their associated challenges and barriers are best acknowledged through discussions and dialogue with various stakeholders.

## Introduction and background

Epilepsy is one of the most common neurological diseases with incidence ranging from 0.5% to 2% in different sittings [1,2]. The World Health Organization (WHO) estimated that nearly 80% of this burden is borne by resource-poor countries where even conventional electroencephalogram (EEG) coverage is dramatically short [3,4].

Many patients initially diagnosed with epilepsy are found not to have it. However, some studies indicate that almost 30% of individuals referred to comprehensive epilepsy centers for refractory epilepsy experience non-epileptic events. These include psychogenic events, syncope, sleep disorders, movement disorders, and migraines. For accurate diagnosis, prolonged video EEG monitoring, lasting from several days to weeks (depending on the specific needs and circumstances of each patient), is necessary, as conducted in epilepsy monitoring units (EMUs) [5,6]. EMUs' main indications are spells' characterization, seizure localization, anti-seizure medication (ASM) adjustment, and epilepsy surgery evaluation and planning [7,8].

However, the conventional EEG approach in EMUs has its obstacles; several studies have undertaken the exploration of challenges relevant to EMUs within the existing literature. Some have discussed technical challenges related to EEG itself [9], challenges related to infants' behaviors and neonatal EEG monitoring difficulties [10,11], or challenges related to artificial intelligence (AI) applications in EMUs [12,13]; their related findings are beyond the scope of the current study. The present article will concentrate on the logistic challenges of EMUs, discussing existing data and limitations and offering suggestions for future planning to enhance the utilization of existing technology.

## Review

Essential components and considerations in establishing EMUs

An EMU requires dedicated space within a hospital, ideally with intensive care unit (ICU) or emergency department (ED) access for managing possible incidents like status epilepticus and falls. While specific policies and procedures for EMUs lack universal guidelines, they should be developed based on international recommendations, expert opinions, and the resources available within the EMU.

Staffing in an EMU is essential and multifaceted. Neurologists specializing in epilepsy and trained in EEG are crucial for accurate diagnosis and treatment planning. Neurosurgeons with epilepsy surgery training to perform epilepsy surgeries for candidate patients and nurses with epilepsy management training play an important role in monitoring and managing patients experiencing seizures. Neurophysiology technicians are responsible for performing and analyzing EEG tests. Neuropsychologists contribute significantly by assessing cognitive functioning and evaluating the risk of cognitive decline post-epilepsy surgery. Lastly, coordinators are important in managing patient care, coordinating with medical staff, handling resources and data, and ensuring the smooth operation of the unit.

The EMU contains specialized medical equipment like video EEG machines and accompanying software which are crucial for continuous and comprehensive patient monitoring. These systems are designed to capture both video and EEG data, often featuring a trigger button for event marking and enhanced analysis. Advanced models can facilitate ambulatory monitoring, allowing data transfer via WiFi.

Furthermore, a data management system forms the cornerstone of an EMU, encompassing efficient data transfer, storage, and retrieval for ongoing analysis. This setup is integral to the effective functioning of an EMU, ensuring timely and precise epilepsy management.

Challenges related to the establishment of EMUs

A shortage in EMUs leads to long waitlists and admission delays, increasing the risk of complications. Data related to the availability of EMUs and their quantity as well as their associated challenges and drawbacks in different sittings is generally scarce.

India has at least five million people with active epilepsy and over 500,000 potential candidates for epilepsy surgery [14]. However, Baheti and colleagues indicated that there are only 12 EMUs in the country, that healthcare facilities are fragile and inefficient, and that the infrastructure, financial, human, and material resources in the health sector are unequally distributed among people in the different economic strata within India [3]. Supporting this, it was previously emphasized that some challenges are unique to low- and middle-income countries; EEG equipment from major suppliers, when imported, arrives at excessive costs to medical or research institutions [15].

In Saudi Arabia, a study conducted by Al-Attas in 2023 indicated a shortage of EMUs and concluded that there are only four "well-established" EMUs in Riyadh City, the capital, with capacity ranging from three to five beds in each unit; this capacity is considered low when compared to almost seven beds per EMU as a mean in a meta-analysis synthesized by data gathered from 25 different countries conducted previously [16]. The author further points to almost 75% bed occupancy rate and that only up to 500 patients are monitored annually in Riyadh City. Moreover, they indicated that authorizing and providing funds for EMU establishment is time-consuming. Furthermore, they highlight a conflict in terms of policy and regulations between different stakeholders, without explicitly delineating specific entities [17].

Another study conducted earlier (2011) by Aljafen and colleagues also in Saudi Arabia indicated that there are 11 EMUs in the country. The average waiting time calculated by all units ranges from two up to 52 weeks, and the percentage of patients coming from an outside region to EMU examination ranges from 5% up to 80% in different locations [18].

Although there is no clear data, to our knowledge, on the quantity of EMUs in the United Kingdom, a study conducted in 2017 aimed at identifying current practices and recommendations for EMUs recruited 32 units: 22 adult EMUs, three pediatric EMUs, and seven serving both. These units provide services for almost 630,000 patients, according to the Epilepsy Research Institute UK. The ease of access to necessary resources and infrastructure likely facilitated EMU establishment, in contrast to India, where such resources are less accessible despite having the highest epilepsy burden. However, the number of EMU beds varies across units, with no set standard per EMU and each unit having between one and seven beds [19].

Healthcare staffing challenges

One challenge in the healthcare sector is the lack of specialized personnel which is not limited to epilepsy services. It is known that the monitoring process, while clinically effective, is labor-intensive and limited resources of numerous roles have been reported in several studies; shortages in EMU manpower such as adult as well as pediatric epileptologists, qualified nurses, and EEG technologists have been reported in different sittings [17,20].

In a study conducted in Saudi Arabia, it was indicated that although fully equipped EMUs are available, they are underutilized considering the number of admitted patients as well as the number of epilepsy surgeries performed annually. A possible reason for such underutilization, they declare, is the lack of supportive services, such as the limited number of EMU technicians. This study indicated that in Dammam, the capital city of the Eastern region, there were only three EMU technicians, while only one EMU technician was available in a unit in the Western region [18].

Moreover, in the study titled "Epilepsy monitoring unit practices and safety among NAEC epilepsy centers: A census survey," the reports of 341 pediatric or adult center directors were analyzed, and the ratio of EEG technologists or nurses to EMU beds was reported to vary according to time of day; 68% of respondents reported a 1:4 ratio of EMU EEG technologists to beds during "regular working hours." Moreover, 75% of nurse coordinators in EMUs under study do not possess neuroscience certification, while 37% of participants did not have an EMU nurse coordinator in the first place. Furthermore, they also highlighted that 12% of monitor watchers, individuals trained to observe and acutely report or respond to seizures, safety concerns, or patient emergencies in the EMU, had restricted access to a physician at specific times and that coverage when leaving a station was not reported by 43% of respondents. To highlight the need for coordination and the smoothness of workflow, the authors indicated that monitor watching among respondents was 61% performed by personnel outside the EMU, including watchers outside the healthcare system in 7% or by a remote monitoring company in 5% [20].

Inside or outside the healthcare facility, these remote monitoring methodologies emphasize a very practical resolution for the healthcare workforce, minimizing the reported staff shortage by introducing telemedicine applications; operating within a tele-EEG network is advised previously for the neurophysiologist to analyze quickly and on demand any significant changes to the EEG signals [21].

Besides, several studies have suggested that non-EEG experts such as bedside ICU personnel can be trained in the acquisition and troubleshooting of EEGs, as well as screening uninterrupted raw EEG for seizure detection purposes [22,23].

EMU safety challenges

Patient safety in EMUs revolves around the risks and sequelae of epileptic seizures, particularly when ASMs are withdrawn to increase the likelihood of capturing seizure attacks. Several original studies and systematic reviews are available in the literature addressing the safety of patients in EMUs and the adverse events associated [24-29]. Ryvlin and colleagues categorized adverse events in EMUs as follows: injuries and falls, cardiac arrhythmias, psychosis, status epilepticus, as well as sudden unexpected death in epilepsy (SUDEP) [30].

The American Epilepsy Society (AES) and the European Epilepsy Monitoring Unit Association conducted studies about adverse events in EMUs [27,31]; they shared comparable findings about the need for EMUs to identify and address potential safety risks. It is indicated that almost 50% out of 70 EMUs who responded to the AES survey reported falls or post-ictal psychosis in the preceding 12 months, while several centers in their study reported falls with concussion or death [27]. Moreover, 7% of centers have encountered a cardiac arrest and 3% death within the past 12 months [27]. The European study on the other hand included 48 EMUs, located in 18 different European countries. All responding EMUs experienced a wide range of seizure-related adverse events; although they reported varying incidences among EMUs, falls and status epilepticus were reported in up to 10% of patients [31].

Furthermore, tens of studies evaluated and assessed different aspects related to safety issues in EMUs. They have been comprehensively reviewed in the study of Rheims and Ryvlin [32], and their review indicated that status epilepticus was reported in 0-3.5% of patients admitted to EMUs; most of them suffered from non-convulsive status epilepticus, whereas convulsive status epilepticus was unusual. Moreover, falls were reported in 1-20% of patients in their included studies, and though most of them led to minor injuries, severe head injury with an acute epidural hematoma requiring immediate emergency surgery, compression fractures of lumbar vertebrae and vertebral fracture, cervical spine injury, and thoracic spine compression have all been reported after a seizure-related fall. Moreover, psychiatric adverse events were reported in 5% of patients; post-ictal psychoses were the most frequent psychiatric adverse event, and half of these patients with post-ictal psychiatric manifestations required hospitalization in a psychiatry department. Rheims and Ryvlin further indicated that cardiac and/or respiratory complications were reported among 2.75%, and they emphasized that injuries were more frequent in patients with a history of injuries (20%) than in those without (3%), patients who experienced vertebral fractures suffered from osteopenia, and patients who suffer from psychiatric complications have a specific history of psychiatric comorbidity (17%) compared to psychiatric complication incidence among patients without such history (1%). Also, the occurrence of status epilepticus was observed in 10% of patients with a previous history of status epilepticus compared to 2% of those with no such history [32]. Consistent with this finding, there is documentation of a case involving a six-year-old male harboring a confirmed SCN1A mutation, associated with Dravet syndrome, and another individual with a history of seizure clustering and post-ictal psychosis; both have exhibited post-ictal psychosis as an EMU adverse event [33].

It is worth noting that psychiatric challenges during EMU admission normally arise due to limited activity and mobility, coupled with medication adjustments and the aim to capture multiple seizures, leading to physical and psychological stressors in addition to post-ictal changes. Long-duration admissions may become tedious, prompting some patients to request discharge against medical advice (DAMA). Moreover, privacy concerns are significant, especially if patients are admitted to shared rooms and bathrooms, alleviated by providing private accommodations. Effective communication from clinic visits to discharge is vital to address patient concerns and set expectations, facilitating proper management and minimizing psychiatric events.

Sauro and colleagues conducted a review in 2016 in their study titled "Quality and safety in adult epilepsy monitoring units: A systematic review and meta-analysis" [16]; 135 studies representing 181,823 patients with epilepsy from 25 different countries were introduced in their review. A noteworthy constraint to consider in the interpretation of their findings is the absence of a minimum threshold concerning the publication dates of studies eligible for inclusion; studies spanning from 1968 to 2016 were included. Consequently, the aggregated estimates synthesized might be inflated due to the incorporation of older studies within each meta-analysis; the disparities in safety, monitoring technology, and quality between the era of 1986 and contemporary times suggest a potential overestimation of the findings. Nevertheless, many of their observations align with other documented findings; however, the calculated overall pooled proportion of all adverse events was reported as 7%, while a much lower estimate of 2% was recently reported (2020) in a study based on a total sample size of 1062 admissions [33]. The frequently reported adverse events in their review were the following: falls, seizure-related injury, status epilepticus, medication-related adverse events, seizure clusters, post-ictal psychosis, and cardiorespiratory complications. Three studies in their review highlighted possible risk factors associated with having an adverse event: being out of bed, lack of staff response, being off-camera, a history of seizure clusters, and seizure frequency prior to admittance [16]. Similarly, Cox and colleagues highlighted falls as the most common complication, comprising 50% of all adverse events. They reported 10 cases (1%) among participants, of which six were categorized as non-injurious [33].

In a study titled "Incidence and mechanisms of cardiorespiratory arrests in epilepsy monitoring units (MORTEMUS): a retrospective study" [30], the authors conducted an international survey and detailed review of EMU data specifically from monitored SUDEP cases; incidents characterized by severe respiratory compromise or cardiac arrhythmia, which did not result in mortality, were classified as near SUDEP. They reported 16 SUDEP and nine near SUDEP from a total of 147 units. Most importantly, and to emphasize the tremendous need for dedicated care with prompt seizure interventions during these times, they highlighted that 14 out of 16 SUDEP cases occurred at night and that the reported time to cardiopulmonary resuscitation (CPR) from seizure end in the SUDEP cases was 13 minutes in one case, and considerably longer or not at all in the other 15 cases, compared to less than one minute to CPR in most of the near SUDEP cases. Nevertheless, they concluded that it is still not possible, on accessible evidence, to assess or estimate a SUDEP risk on the EMU.

Additionally, the International Mortality in EMUs (MORTEMUS) study aimed at quantifying the risk of SUDEP over many EMUs in several countries; their findings showed that the incidence of SUDEP among adults in EMUs is almost five per 1000 patient-years (95% CI=2.6-9.2) and that SUDEP and near SUDEP were encountered by more than 10% of all EMUs surveyed in the study [30].

Moreover, several studies highlighted a less serious safety issue of prolonged EEG monitoring; skin irritation or injuries at the EEG electrode sites are reported to occur in almost 8% up to 27% of patients, depending on the duration of the monitoring period [34]. Furthermore, the study conducted by Ouchida and colleagues in 2019 indicated a much higher prevalence, as most participants (82%) had skin irritation or injury at the electrode sites [35]. Possible mechanisms have been previously described as causes; mechanical injury can occur during the process of skin preparation, and extended direct pressure from the electrodes on the head during the monitoring or at the time of electrode removal and chemical injury can also occur during the process of applying conductive agents, causing irritation or contact dermatitis [35].

Ouchida and colleagues in 2020 conducted a non-randomized study investigating different types of cream and the related consequences in skin injury at the site of EEG electrodes; they indicated that skin injury/inflammation was significantly less using a combination of Ten20® with Tensive® gel as the conductive material [36].

Bagić and colleagues spotted light about some risky behaviors; out of 341 EMU directors in their study, 35% declared that patients were not supervised whenever moving around the room, and bedside commodes were provided in 49% of respondents, with 74% continuing EEG recording while patients are using the restroom. Bagić and colleagues further indicated in their study that 49% of respondents (out of 341 EMU directors) required a non-medical observer for an agitated patient and 45% engaged a psychiatrist for assistance, while 34% did not have a standard protocol for patient agitation. Moreover, intravenous (IV) catheters were placed among 53% of patients who had ASMs reduced [20].

Lastly, Cox and colleagues, in their study titled "Epilepsy monitoring units can be safe places; a prospective study in a large cohort," emphasized that there was a significant association between the type of recording (diagnostic versus event recording) and the occurrence of adverse events [33].

It is useful to highlight that seizure complications are not limited to the EMU but pose a risk for any patient with seizures, particularly if they are uncontrolled. Being in an EMU makes it more likely for these complications to be documented and observed; this doesn't imply that the EMU itself is inherently risky. Rather, it underscores that seizure complications that typically occur outside EMUs can also happen within them. Therefore, there should be heightened caution to prevent these complications, and if they do occur, they must be managed appropriately.

Patient-related challenges

In some cases, predominantly if the primary purpose of the EMU admission is to assess treatment response or evaluate seizure frequency and severity rather than diagnose seizure, patients may be required to continue their regular medication regimen all the way through the monitoring period. It is not uncommon in clinical practice to encounter patients who report experiencing multiple seizure attacks prior to EMU admission, only to exhibit none during their hospital stay. This phenomenon presents a potential risk of non-adherence to ASMs among patients. While empirical investigations specifically focusing on non-adherence in the context of EMUs are currently lacking in the literature, to our knowledge, it is well established that ASMs may elicit adverse effects preventing patients from being compliant [37-39].

Prospects for future developments

Identifying Barriers to Establishing EMUs

Identifying barriers to establishing EMUs can be further explored through surveys and workshops conducted within each country; several similar studies have been reported previously [14,19,25,27,31]. These methodologies provide a localized platform for stakeholders to discuss challenges and the possibility of regulatory reform. In some cases, conflicts may arise due to outdated, ineffective, or complex regulations and accreditation standards. Stakeholders can also share insights and, most importantly, propose solutions relevant to the unique sociocultural, regulatory, and healthcare system dynamics of the country. This approach is proposed to enable more focused and actionable strategies to address barriers to establishing EMUs. On the other hand, bringing stakeholders from different regions facilitates the exchange of knowledge, experiences, and perspectives across varied geographical settings, allowing for the identification of common challenges as well as unique obstacles specific to geographical settings. Furthermore, these workshops promote networking and partnerships among stakeholders, laying the groundwork for coordinated efforts and collective action to overcome barriers and improve access to EMUs worldwide.

Conduction of Studies Employing Larger Sample Sizes

Studies with larger sample sizes have the potential to afford more statistically robust and generalizable conclusions regarding the challenges. By including more patients with epilepsy, these studies can capture a broader array of experiences and outcomes, increasing the consistency and validity of the results. Additionally, larger sample sizes allow for more comprehensive analyses of potential risk factors, subgroup differences, as well as unusual adverse events in EMUs. This is believed to dramatically enhance the precision and accuracy of safety risk assessments, enabling more informed decision-making in clinical practice and policy development.

Utilization of AI in EMUs

The proliferation of AI applications has become widespread, permeating various domains and sectors. Regarding epilepsy, numerous studies that examined the specificity and sensitivity of seizure detection using AI-designed algorithms have been reported and were comprehensively reviewed [12,13]. Similar applications are to be appreciated, for instance, in predicting safety risks in EMUs. This is approached by leveraging advanced algorithms to analyze vast datasets of patient information and adverse event patterns. Integrating real-time monitoring data, patient history, and other sociocultural and environmental factors, AI-driven predictive models can provide timely alerts to healthcare providers, enabling proactive interventions and reducing the risk of adverse events in EMUs. Furthermore, AI algorithms are known to be continuously learning and adapting from new data, refining their predictive capabilities over time, and as a result enhancing patient safety in EMUs. One critical issue to be addressed is that algorithms need to be trained and tested on a large amount of EEG data comprising a complete monitoring period and including any kind of artifacts as well as non-ictal physiological and abnormal EEG patterns, as previously described [40,41]. Moreover, further attempts to develop patient-specific algorithms, designed to improve sensitivity and selectivity for individual patients, are highly valued; they have been previously attempted among patients with epilepsy [42-44].

Data Management and Integration

Integrated data platforms enable healthcare providers to access comprehensive patient records, fostering accurate diagnosis, individualized treatment approaches, and interdisciplinary collaboration within EMUs. By centralizing data storage and facilitating real-time access to patient records, EMUs can enhance communication, coordination of care, as well as quality improvement initiatives. Moreover, integrated data repositories support research endeavors and innovation by facilitating data sharing, analysis, and collaboration opportunities. Furthermore, it is noteworthy to acknowledge that several proposed avenues for future development, as mentioned above, such as the utilization of AI-powered algorithms or the execution of large cohort studies, inherently necessitate a substantial amount of available datasets.

Current study limitations

The current study, due to the scarcity of research articles, does not provide evidence synthesized by calculating pooled prevalence estimates through a meta-analysis approach to provide better judgment regarding the safety risks or adverse events in EMUs in different geographical settings. Such data may directly act as cornerstones in designing containment strategies or preventive measures. Moreover, a systematic approach in including relevant studies was not conducted; instead, several original research articles as well as reviews related to the subject were comprehended, reviewed, and analyzed. Furthermore, the number of studies addressing the primary objective of the present review is limited, and they originate from a restricted geographical scope.

## Conclusions

Regarding healthcare-related challenges, several studies have reported shortages of adult and pediatric epileptologists, qualified nurses, as well as EEG technologists. Moreover, injuries and falls, psychosis, status epilepticus, and SUDEP have been stated to be the most frequent safety challenges in EMUs. Furthermore, enhancements to mitigate logistical and healthcare system-related barriers in EMUs include the implementation of large cohort studies and the utilization of AI for the identification and categorization of specific risks among EMU admissions, while the scarcity of EMUs and their associated challenges and barriers are best acknowledged through discussions and dialogue with various stakeholders.
